# Supplementary material for: Characterization of Botanical Origin of Italian Honey by Carbohydrate Composition and Volatile Organic Compounds (VOCs)
Source: Foods. 2022 Aug 13;11(16):2441. doi: 10.3390/foods11162441 (PMC9407073; doi:10.3390/foods11162441)
Supplement: Supplementary file 1 [file foods-11-02441-s001.zip › foods-1820003-supplementary.pdf]

Supplementary materials

Characterization of Botanical Origin of Italian Honey by Carbohydrate Composition and Volatile Organic Compounds (VOCs)

Raffaello Tedesco [1,2], Elisa Scalabrin [1,3], Valeria Malagnini [2], Lidija Strojnik [4], Nives Ogrinc [4] and Gabriele Capodaglio [1,*]

[1] Department of Environmental Sciences, Informatics and Statistics, University of Venice, Ca' Foscari, Via Torino 155, 30172 Venice Mestre, Italy
[2] Centro Ricerca e Innovazione, Fondazione Edmund Mach (FEM), Via E.Mach, 1, San Michele all'Adige, 38010 Trento, Italy
[3] National Research Council, Polar Science Institute, Via Torino 155, 30172 Venice Mestre, Italy
[4] Department of Environmental Sciences, Jožef Stefan Institute, 1000 Ljubljana, Slovenia
* Correspondence: capoda@unive.it

Supplementary Table

Table S1. Eighty volatile organic compounds identified in floral honey samples by HS-SPME-GC/MS, reporting retention time and molecular weight of each aromatic compound.

Compound	Retention time (min)	Molecular weight
Octane	2.1	114
Ethyl Acetate	2.6	88
Butanal, 3-methyl-	2.9	86
Ethanol	3.5	46
2,3-Butanedione	3.7	86
Pentanal, 3-methyl-	4.6	100
Propanal, 2-methyl-	6.8	72
Dodecane	8.6	170
D-Limonene	8.7	136
γ-Terpinene	10.4	136
o-Cymene	11.4	134
Octanal	12.2	128
2-Nonanone	16.1	142
1,3,8-p-Menthatriene	16.2	134
Nonanal	16.3	142
Tetradecane	16.6	198
p-Mentha-1,5,8-triene	17.4	134
trans-Linalool oxide (furanoid)	18.2	170
Acetic acid	19.1	60
Furfural	19.2	96
Decanal	20.5	156
Pentadecane	20.7	212
Benzaldehyde	21.3	106
Lilac aldehyde A	22.1	168
Lilac aldehyde C	22.5	168
Linalool	22.7	154
Lilac aldehyde B	22.9	168
Propanoic acid, 2-methyl-	23.6	88
Lilac aldehyde D	23.8	168
Terpinen-4-ol	24.5	154
Hexadecane	24.7	226
1,5,7-Octatrien-3-ol, 3,7-dimethyl- (Hotrienol)	25.1	152
Butanoic acid	25.8	88
Benzeneacetaldehyde	26.0	120
Butanoic acid, 2-methyl- and Butanoic acid, 3-methyl-	27.4	102
Terpineol	28.3	154
2-Furanmethanol	28.6	98
2,6-Dimethyl-1,3,5,7-octatetraene, E,E-	30.6	134
1-Nonanol	30.8	144
Pentanoic acid, 3-methyl-	31.8	116
Hexanoic acid	33.7	116
1-Decanol	34.8	158
Nonadecane	35.8	268
Lilac alcohol A	36.8	170
Creosol	37.2	138

Table S1 (Continued). Eighty volatile organic compounds identified in floral honey samples by HS-SPME-GC/MS, reporting retention time and molecular weight of each aromatic compound.

**Compound**	**Retention time (min)**	**Molecular weight**
Benzylalcohol	37.6	108
Hexanoic acid, 2-ethyl-	38.1	144
Heptanoic acid	38.2	130
3,7-Octadiene-2,6-diol, 2,6-dimethyl-	38.6	170
Phenylethylalcohol	40.8	122
Cinnamaldehyde (E)	42.0	132
Octanoic acid	44.8	144
Heneicosane	46.9	296
m-Guaiacol	47.8	124
p-Cymene	47.9	134
Caprolactam	48.7	113
Eugenol	48.9	164
3-Phenylpropanol	49.1	136
Nonanoic acid	49.4	158
Thymol	50.1	150
n-Decanoic acid	52.5	172
Eicosane	53.2	282
Heptadecane	53.3	240
Octadecane	53.3	254
Geranic acid	54.1	168
Diethyl Phthalate	54.4	222
Benzoic acid	55.9	122
2,7-Octadiene-1,6-diol, 2,6-dimethyl-	56.2	170
Dodecanoic acid	57.2	200
Benzeneacetic acid	58.5	136
5-Hydroxymethylfurfural	58.6	126
Dibutyl phthalate	60.7	278
Tetradecanoic acid	60.9	228
trans-Cinnamic acid	63.7	148
n-Hexadecanoic acid	64.6	256

Table S2. Data of mean area value, standard deviation, and relative standard deviation (RSD%) of 24 identified volatile organic compounds calculated on seven replicates (n=7) of a multifloral honey.

Volatile compound	Mean	Std Dev	RSD %
Octane	3753826	677092	18
γ-Terpinene	3168509	114746	4
Octanal	2392483	258763	11
2-Nonanone	2235022	143391	6
Nonanal	16689855	1406944	8
Acetic acid	7702118	344264	4
Furfural	57421079	3992440	7
Decanal	5206367	240636	5
Benzaldehyde	1,91E+08	15223791	8
Lilac aldehyde C	2618819	227678	9
Linalool	11761973	1201352	10
1,5,7-Octatrien-3-ol, 3,7-dimethyl- (hotrienol)	1,6E+08	9594495	6
Benzeneacetaldehyde	24809299	763505	3
Terpineol	9029434	698375	8
Heptanoic acid	7228056	598734	8
Phenylethyl alcohol	19807629	846223	4
Octanoic acid	49753568	2011451	4
Nonanoic acid	60961622	5071476	8
Thymol	35527182	1191042	3
n-Decanoic acid	20925199	757184	4
Geranic acid	11508485	343189	3
Benzoic acid	37680359	1778475	5
Dodecanoic acid	8296098	918573	11
Tetradecanoic acid	2778336	441329	16

Table S3. Melissopalynological analysis of the floral honey samples; principal (>45%) and/or accompanying pollen (15-45%), and important minor pollen types (3-15%).

Sample	Floral type	Family	Principal pollen (>45%) and/or accompanying important pollen (15-45%)	Important minor pollen (3-15%)
M36	Multifloral	Ericaceae	42.50%	
		Salicaceae	*Salix* (38.0%)	
		Hippocastanaceae		*Aesculus* (5.7%)
		Rosaceae		*Malus/Pyrus* (3.8%)
		Asteraceae		*T-Form* (3.5%)
M37		Ericaceae	36.50%	
		Fabaceae		*Trifolium repens* (12.8%)
		Asteraceae		*T-Form* (9.1%)
		Rosaceae		*Rubus* (6.8%)
		Apiaceae		5.70%
		Rosaceae		4.30%
		Ranunculaceae		*Clematis* (3.1%)
M38		Ericaceae	32.60%	
		Rosaceae	*Rubus* (16.0%)	
		Asteraceae		*A-Form* (9.6%)
				T-Form (3.2%)
		Buddlejaceae		*Buddleja* (8.1%)
		Fabaceae		*Trifolium pretense* (4.2%)
				Trifolium repens (3.7%)
		Lauraceae		3.20%
		Rosaceae		*Malus/Pyrus* (3.0%)
M39		Ericaceae	62.80%	
		Fagaceae		*Castanea sativa* (12.1%)
		Apiaceae		*A/H-Form* (4.0%)
		Ranunculaceae		*Clematis* (3.5%)
		Boraginaceae		*Echium* (3.3%)
		Fabaceae		*Trifolium repens* (3.3%)
M41		Ericaceae	55.60%	
		Apiaceae		*A/H-Form* (8.8%)
		Asteraceae		*T-Form* (3.8%)
		Rosaceae		*Rubus* (3.8%)
		Salicaceae		*Salix* (3.2%)
		Scrophulariaceae		*Rhinanthus* (3.2%)
M42		Ericaceae	35.50%	
		Apiaceae		*A/H-Form* (10.9%)
		Rosaceae		*Rubus* (8.1%)
				Others (5.5%)
		Fabaceae		*Trifolium repens* (7.3%)
				Lotus (4.5%)
		Polygonaceae		*Fagopyrum esculentum* (3.3%)
		Scrophulariaceae		*Rhinanthus* (3.1%)

Table S3 (continued). Melissopalynological analysis of the floral honey samples; principal (>45%) and/or accompanying pollen (15-45%), and important minor pollen types (3-15%).

M44	Fagaceae	*Castanea sativa* (81.1%)	
	Salicaceae		*Salix* (10.2%)
	Rosaceae		*Malus/Pyrus* (3.0%)
M3-18	Ericaceae	70.90%	
	Rubiaceae		8.70%
	Fabaceae		*Trifolium repens* (4.8%)
M5-18	Fagaceae	*Castanea sativa* (81.1%)	
	Vitaceae		*Parthenocissus* (3.0%)
M6-18	Fagaceae	*Castanea sativa* (81.1%)	
	Asteraceae		*A-Form* (4.1%)
M7-18	Fagaceae	*Castanea sativa* (78.4%)	
	Ericaceae		14.10%
	Tiliaceae		*Tilia* (3.1%)
M9-18	Fagaceae	*Castanea sativa* (75.6%)	
	Tiliaceae		*Tilia* (3.1%)
M16-18	Ericaceae	55.50%	
	Rosaceae		*Rubus* (5.7%)
			Fragaria-Potentilla (4.8%)
	Tiliaceae		*Tilia* (4.4%)
	Ranunculaceae		*Clematis* (3.5%)
	Scrophulariaceae		*Rhinanthus* (3.5%)
M19-18	Ericaceae	25.30%	
	Rosaceae		*Rubus* (13.6%)
			Malus/Pyrus (5.1%)
	Fabaceae		*Onobrychis* (9.6%)
	Salicaceae		*Salix* (9.6%)
	Ranunculaceae		*Clematis* (7.1%)
	Apiaceae		4.00%
M20-18	Ericaceae	80.40%	
	Rosaceae		*Rubus* (11.1%)
M21-18	Ericaceae	80.10%	
	Rosaceae		*Rubus* (5.7%)
	Fabaceae		*Tripholium repens* (4.1%)
M23-18	Fagaceae	*Castanea sativa* (73.7%)	
	Tiliaceae	*Tilia* (16.5%)	
	Rosaceae		*Rubus* (3.4%)
M46 C	Fagaceae	*Castanea sativa* (19.9%)	
	Fabaceae	*Tripholium repens* (19.2%)	
		Robinia (3.5%)	
	Scrophulariaceae	*Verbascum* (18.5%)	
	Rosaceae		*Rubus* (11.1%)
			Others (4.5%)
	Ranunculaceae		*Clematis* (4.2%)
	Rubiaceae		3.80%

Table S3 (continued). Melissopalynological analysis of the floral honey samples; principal (>45%) and/or accompanying pollen (15-45%), and important minor pollen types (3-15%).

M46 P	Fagaceae	*Castanea sativa* (19.3%)	
	Scrophulariaceae	*Verbascum* (17.9%)	
			Rhinanthus (6.1%)
	Fabaceae	*Tripholium Repens* (15.2%)	
		Robinia (3.4%)	
	Rosaceae		*Rubus* (10.8%)
			Others (4.4%)
	Caesalpiniaceae		*Gleditsia* (4.1%)
	Rubiaceae		3.70%
M46 D	Rosaceae	*Rubus* (21.3%)	
			Malus/Pyrus (3.7%)
	Fagaceae	*Castanea sativa* (18.4%)	
	Scrophulariaceae		*Rhinanthus* (12.5%)
	Fabaceae		*Tripholium Repens* (11.4%)
			Robinia (4.4%)
	Ranunculaceae		*Clematis* (8.1%)
	Cornaceae		*Cornus* (4.0%)
	Rubiaceae		3.70%
	Salicaceae		*Salix* (3.3%)
M47C	Fagaceae	*Castanea sativa* (41.2%)	
	Rosaceae		*Malus/Pyrus* (11.1%)
			Prunus (4.6%)
			Others (3.8%)
	Salicaceae		*Salix* (6.8%)
	Fabaceae		*Robinia* (5.7%)
	Cruciferae		4.30%
	Rubiaceae		4.10%
	Asteraceae		*T-Form* (3.3%)
M47 P	Fagaceae	*Castanea sativa* (41.3%)	
	Rosaceae		*Prunus* (8.6%)
			Malus/Pyrus (7.6%)
			Rubus (6.3%)
	Rhamnaceae		5.80%
	Rubiaceae		4.60%
	Cruciferae		3.50%
	Asteraceae		*T-Form* (3.3%)
M47 D	Fagaceae	*Castanea sativa* (50.4%)	
	Rhamnaceae		6.60%
	Rosaceae		*Malus/Pyrus* (6.9%)
			Rubus (6.4%)
			Prunus (5.6%)
	Rubiaceae		3.70%
	Fabaceae		*Robinia* (3.4%)

Table S3 (continued). Melissopalynological analysis of the floral honey samples; principal (>45%) and/or accompanying pollen (15-45%), and important minor pollen types (3-15%).

M22-18		Rosaceae	*Rubus* (16.6%)	
			Others (10.3%)	
				Prunus-Gr (3.3%)
				Malus/Pyrus (5.5%)
		Fabaceae		*Robinia* (14%)
		Arecaceae		*Camerops* (11.1%)
		Simaroubaceae		*Ailanthus* (11.1%)
		Hippocastanaceae		*Aesculus* (5.5%)
		Apiaceae		*A-H Form* (3.7%)
		Salicaceae		*Salix* (3.7%)
A1-18	Acacia	Fagaceae	*Castanea sativa* (46.3%)	
		Fabaceae	*Robinia* (22.4%)	
		Rosaceae	*Rubus* (16.1%)	
A11-18		Fagaceae	*Castanea sativa* (46.6%)	
		Fabaceae	*Robinia* (17.2%)	
				Tripholium Repens (3.4%)
		Rosaceae		*Rubus* (7.8%)
		Scrophulariaceae		*Rhinanthus* (5.0%)
		Rhamnaceae		3.80%
A28-18		Fabaceae	*Robinia* (19.8%)	
		Rosaceae	*Rubus* (19.4%)	
			Malus/Pyrus (19.0%)	
		Fagaceae	*Castanea sativa* (14.1%)	
		Ebenaceae	*Diospyros* (4.6%)	
AD43	Apple-Dandelion	Fagaceae	*Castanea sativa* (32.2%)	
		Asteraceae	*T-Form* (23%)	
		Salicaceae	*Salix* (19.7%)	
		Rosaceae	*Malus/Pyrus* (18.1%)	
AD45		Fagaceae	*Castanea sativa* (49.5%)	
		Asteraceae		*T-Form* (14.0%)
		Rosaceae		*Malus/Pyrus* (12.1%)
		Hydrophyllaceae		*Phacelia* (3.8%)
		Simaroubaceae		*Ailanthus* (3.3%)
		Vitaceae		*Parthenocissus* (3.0%)
AD25-18		Rosaceae	*Malus/Pyrus* (22.6%)	
				Rubus (11.6%)
		Vitaceae		*Parthenocissus* (12.5%)
		Fabaceae		*Robinia* (11.9%)
		Arecaceae		*Camerops* (9.8%)
		Hippocastanaceae		*Aesculus* (4.2%)
R2-18	Rhododendron	Fagaceae	*Castanea sativa* (64.2%)	
		Ericaceae	31.30%	
R4-18		Ericaceae	87.10%	
		Fagaceae		*Castanea sativa* (6.5%)

Table S3 (continued). Melissopalynological analysis of the floral honey samples; principal (>45%) and/or accompanying pollen (15-45%), and important minor pollen types (3-15%).

R14-18		Ericaceae	84.60%	
		Rosaceae		*Rubus* (5.5%)
		Fagaceae		*Castanea sativa* (4.4%)
		Ericaceae	82.60%	
R17-18		Rosaceae		*Rubus* (3.7%)
		Ericaceae	91.00%	
R18-18		Campanulaceae		3.90%
		Ericaceae	91.10%	
R24-18		Fagaceae		*Castanea sativa* (7.2%)
R27-18		Ericaceae	69.60%	
		Fagaceae	*Castanea sativa* (23.0%)	
HD15-18	Honeydew		Honeydew elements (HDE/P=3.13)	
HD26-18			Honeydew elements (HDE/P=3.57)	
HD29-18			Honeydew elements (HDE/P=7.61)	
C40	Chestnut	Fagaceae	*Castanea sativa* (90.0%)	
C8-18		Fagaceae	*Castanea sativa* (98.0%)	
C12-18		Fagaceae	*Castanea sativa* (97.8%)	
C13-18		Fagaceae	*Castanea sativa* (91.7%)	
C52 C		Fagaceae	*Castanea sativa* (97.0%)	
C52 P		Fagaceae	*Castanea sativa* (96.4%)	
C52 D		Fagaceae	*Castanea sativa* (95.1%)	

Supplementary figures

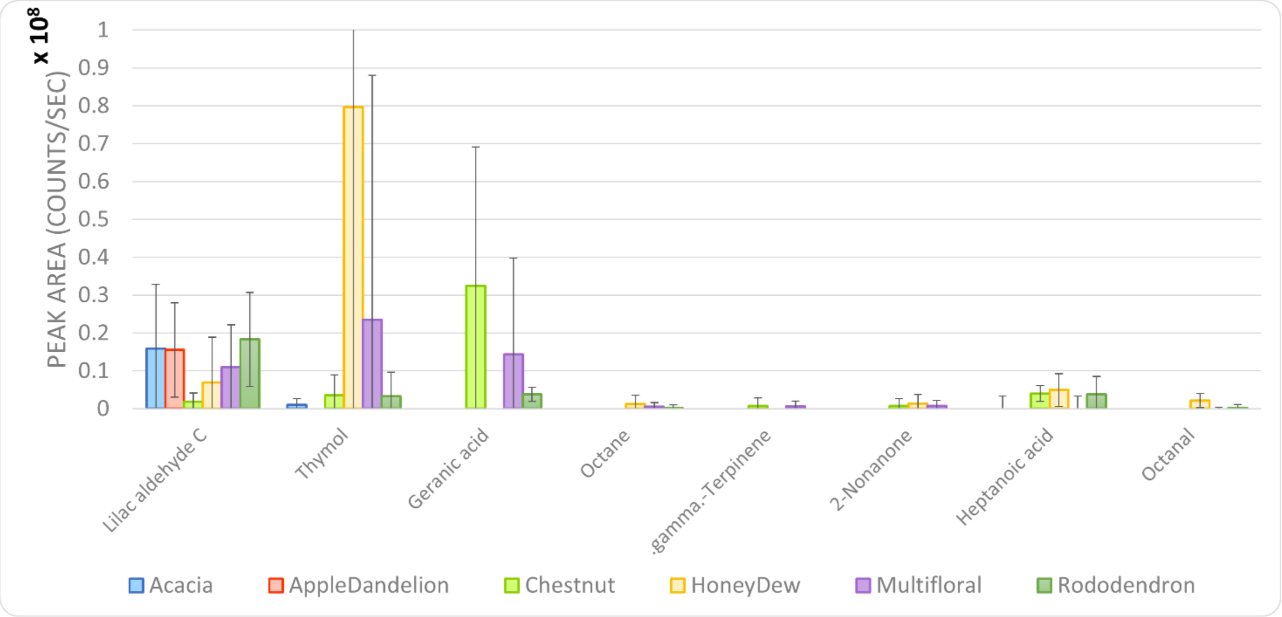

Figure S1 Eight less intense and not statistically significant VOCs in honey samples. Error bars represents standard deviations.
